# Clinical Nursing Management of Adult Patients with Delirium in a Hospital Setting—A Systematic Review

**DOI:** 10.3390/jcm14228113

**Published:** 2025-11-16

**Authors:** Anna Szewczak, Dorota Siwicka, Jadwiga Klukow, Joanna Czerwik-Marcinkowska, Szymon Zmorzynski

**Affiliations:** 1 Department of Anesthesiology Nursing and Intensive Care, Medical University of Lublin, 20-059 Lublin, Poland; 2 I^st^ Department of Anesthesiology and Intensive Care, Medical University of Lublin, 20-059 Lublin, Poland; 3 Institute of Clinical Sciences, Academy of Zamosc, 22-400 Zamosc, Poland; 4 Institute of Biology, Jan Kochanowski University of Kielce, 25-369 Kielce, Poland; 5 Institute of Human Sciences, Academy of Zamosc, 22-400 Zamosc, Poland

**Keywords:** delirium, acute confusional state, nursing care, prevention, nonpharmacological interventions

## Abstract

**Background/Objectives**: Delirium is a common and serious neurocognitive disorder among hospitalised adults, which is associated with prolonged hospital stays, increased treatment costs, and increased mortality. Nurses, as healthcare professionals in constant contact with patients, play crucial roles in the early recognition, prevention, and management of delirium. This systematic review aimed to synthesise the evidence on nursing roles and interventions in the recognition, prevention, and treatment of delirium in hospitalised adult patients. **Methods**: A systematic literature search was conducted in PubMed/MEDLINE, Scopus, and CINAHL/EBSCO for studies published between January 2015 and August 2025. The protocol was registered in PROSPERO. **Results**: Out of the 3791 records identified, 39 met the inclusion criteria. Studies included randomised controlled trials, systematic reviews, and cross-sectional studies conducted in various hospital settings. Key nursing roles included early detection of risk factors, routine use of tools (e.g., CAM, CAM-ICU, 4AT), and implementation of multicomponent nonpharmacological prevention programs for patients with delirium. The evidence regarding the effectiveness of nurse-led interventions has been mixed: several studies reported reductions in delirium incidence and duration with early mobilisation, sleep promotion, cognitive stimulation, pain control and family involvement, whereas others reported no statistically significant effects. **Conclusions**: Routine screening, targeted nonpharmacological interventions, and interdisciplinary collaboration are essential for improving delirium-related outcomes. However, the findings should be interpreted with caution due to differences in the study design, variability in nursing practice across countries, and language restrictions that may have limited the scope of the review. Further high-quality studies are needed to clarify the impact of specific nursing-led strategies on delirium outcomes.

## 1. Introduction

Delirium is an acute neuropsychiatric disorder that results in the loss of the ability to perceive, purposefully recognise stimuli from the environment, process them, and respond appropriately to the information received. Even if a person remains conscious, their cognitive functions undergo qualitative changes [1,2]. In medical classifications (e.g., ICD-10/ICD-11, DSM-5), delirium is defined as an acute, transient disturbance of a person’s consciousness and cognitive function [3,4]. It is a set of symptoms characterised by a sudden change in mental state, developing over a short period of time—from a few hours to a few days, rarely months—with a variable course and a significant impact on the patient’s prognosis [5,6]. The medical literature also describes “persistent delirium,” in which symptoms of cognitive impairment can persist for months or even years after discharge from the hospital. The incidence of delirium among hospitalised patients varies greatly depending on the population, type of ward, and assessment methods used. In general wards, it occurs in approximately 20–30% of patients, whereas in intensive care units, the incidence can reach as high as 70%. In patients who have undergone major surgery or have neurological diseases (e.g., after a stroke), delirium occurs in 25–50% of patients. In the population of older adults treated on an emergency basis, this percentage is usually 10–15% [6,7,8,9]. Delirium is a relatively common complication among hospitalised adults. Its prevalence is influenced by the characteristics of the population, the type of hospital ward, and the diagnostic methods employed [10]. It is estimated to occur in 10–80% of patients [3,6,11,12], particularly in elderly individuals, after stroke, in postoperative patients (20–50% of individuals over 60) [13] and patients treated in intensive care units (ICUs) [3,6,14,15]. Mechanically ventilated patients are at particularly high risk, with delirium occurring in 50–70% of cases [6,7,16]. Delirium is common in individuals who have survived cardiac arrest (CA) [2,17], and is least common among gynecological patients [18].

There are three subtypes of delirium: hyperactive, hypoactive, or mixed [6,13]. Despite its prevalence, delirium often goes unrecognised, especially in its hypoactive form [7,19,20,21], which can resemble fatigue [20], depression, or dementia [22]. Studies indicate that in ICUs and hospital emergency departments, symptoms of delirium remain undiagnosed in 66–84% of cases [3]. Delirium is secondary in nature and most often results from an underlying disease, injury, or external factor. Factors such as age, immobilisation (e.g., due to the presence of catheters or as a result of physical restraint), sleep disorders, malnutrition, the use of sedatives and analgesics, and hospitalisation in an ICU contribute to the onset of delirium [20]. These factors can be divided into predisposing (basic) and triggering (acute) factors [3,6,7]. Effective treatment requires identification of the cause, which is often a combination of several coexisting factors [3,23].

Despite the availability of validated screening tools and international guidelines, the problem of undiagnosed delirium remains relevant. In 2025, updated international guidelines for the prevention and management of delirium were published [24], and in 2023, recommendations for adult patients undergoing surgery were published [25]. Recent studies indicate that although screening tools are available, their use in clinical practice, even in intensive care units, is not standard [26,27,28,29]. Owing to the lack of effective pharmacological prevention methods [16], nonpharmacological strategies play a key role, including nursing activities focused on early diagnosis and prevention of delirium [3]. Owing to their constant contact with patients, they play a key role in observing mental health, implementing preventive procedures, and supporting treatment [30].

Although the clinical aspects, risk factors, and effects of delirium are well described, the contribution and importance of nursing practice in this area remain insufficiently defined. Previous reviews have focused mainly on pharmacological treatment or an interdisciplinary approach, often overlooking the specific responsibilities and activities of nurses in the care of patients with delirium. There is also a lack of synthesis of data on the effectiveness of nursing interventions aimed at early diagnosis, prevention, and treatment of delirium in various hospital settings. This gap justifies the need for a systematic review of nursing roles and interventions in the care of patients with delirium.

The aim of this review is to synthesise evidence from studies published between 2015 and 2025 and to address the role of nurses in the prevention, recognition, and management of delirium in hospitalised patients.

## 2. Methods

This systematic review was conducted in accordance with the PRISMA 2020 guidelines, which provide standardised criteria for conducting and reporting systematic reviews, ensuring transparency and reproducibility. The protocol was registered in the PROSPERO database (registration number: CRD420251143918).

### 2.1. Search Strategies

A systematic literature search was conducted in three databases: PubMed/MEDLINE, Scopus, and CINAHL/EBSCO. The search covered publications from the last 10 years (from 1 January 2015 to August 2025). The search strategy included combinations of the following keywords: (delirium OR confusion OR agitation OR (neurocognitive AND disorder*) OR (cognitive AND impairment) OR (acute AND confusional AND state) OR (acute AND confusion) OR inattention OR (consciousness AND disorder*) OR (cognitive AND disorder*) OR (neuropsychological AND disorder*) OR disorientation) AND ((intensive AND care AND unit*) OR ICU OR Non-ICU OR (critical AND care) OR (acute AND critical) OR (care AND unit) OR hospital* OR “general ward” OR “medical ward” OR “geriatric ward” OR “internal medicine” OR “neurology ward” (perioperative AND care))) AND (((nonpharmacolog* OR environmental OR multicomponent OR package) AND (care OR intervention* OR management OR prevent* OR treat* OR therap* OR assessment OR monitoring OR diagnosis OR role)) AND (nurse* OR nursing OR nurses)). The keywords have been tailored to the specific characteristics of each database. All databases (PubMed/MEDLINE, Scopus, and CINAHL/EBSCO) were last searched in October 2025. No additional sources, registers, or organisational websites were searched beyond these databases.

### 2.2. Inclusion and Exclusion Criteria

The review included studies published between 2015 and 2025 that focused on hospitalised adult patients (≥18 years). Eligible articles assessed nurses’ knowledge of delirium and described nursing activities, the role of nurses, or nursing interventions related to delirium in the care of adult patients in hospitals. Only full-text publications available online or as PDF files on scientific platforms and written in Polish or English were considered.

The review excluded studies focusing solely on pharmacotherapy without the involvement of nursing staff or pediatric populations. Works not available in full text, including conference abstracts, letters to the editor, and comments, were also omitted. In addition, studies concerning patients at home after hospital discharge and records that did not constitute original studies were excluded.

The selection of scientific articles was carried out by verifying titles, abstracts, and full texts via relevant keywords (Table 1). The number of excluded articles was 2460. The most common reasons for exclusion were studies that did not involve nursing staff, studies focusing exclusively on pharmacological treatment, studies without results related to delirium, conference abstracts, and articles not available in full text.

### 2.3. Information on the Included Literature

**Figure 1:**
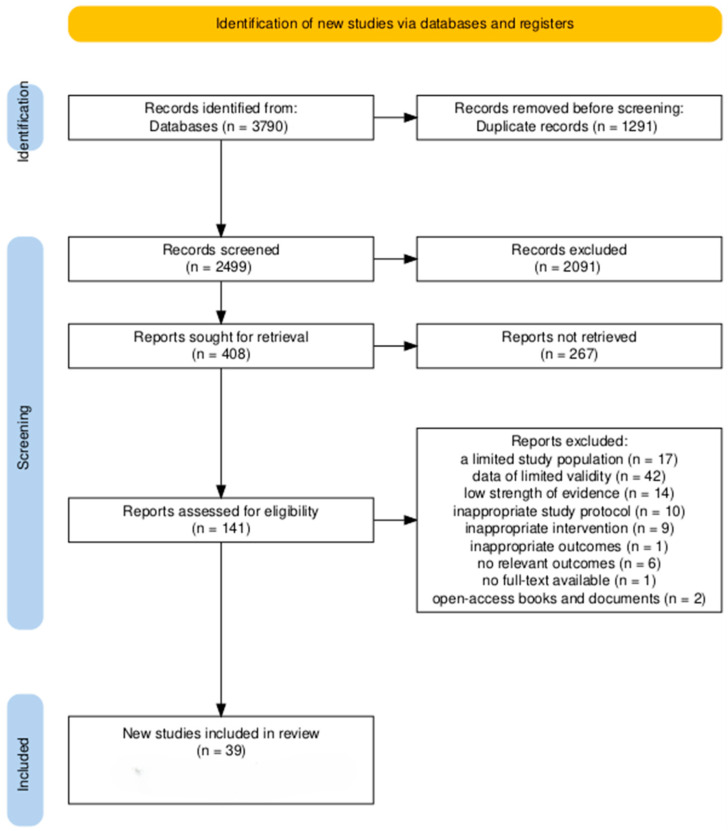
The PRISMA flowchart was created via the tool [31].

The scientific literature search was conducted in the following databases: PubMed/MEDLINE, Scopus, and CINAHL/EBSCO. To increase the precision of the search, database filters were applied, including full-text availability and publication date between 2015 and 2025. Two independent authors reviewed the titles and abstracts of the articles. In cases of discrepancies, the decision was made jointly or with the participation of a third reviewer. The selection process is presented in the PRISMA diagram (Figure 1). The completed PRISMA checklist is provided in the Supplementary Material. All included papers met the predefined inclusion criterion of being available in full text at the time of the review process–either as open-access publications or through institutional access enabling full-text download in PDF format. Therefore, the selection process remained consistent with the eligibility criteria, which focused on full-text accessibility, rather than strictly on open-access licensing status.

### 2.4. Data Extraction

The data were extracted via a standardised form. The following information was extracted from each study: the author and year of publication, the country and setting of the study, the study design, and the characteristics of the population. The data also included a description of the nursing interventions; results related to the recognition, prevention, or treatment of delirium; and the delirium assessment tools applied (e.g., Confusion Assessment Method–CAM; Confusion Assessment Method for the Intensive Care Unit-CAM-ICU; Intensive Care Delirium Screening Checklist-ICDSC). Data extraction was performed independently by two reviewers via a standardised form. Any discrepancies were resolved through discussion or consultation with a third reviewer. The primary outcomes that were analysed included nurses’ knowledge of delirium, their professional role, and nonpharmacological interventions related to the recognition, prevention, and treatment of delirium. All available results within these thematic areas were obtained from each study.

### 2.5. Data Synthesis

Owing to the diversity of study designs, populations, and outcome measures, a narrative synthesis was conducted in four stages: (I) summarising the results of each study in Table 1; (II) analysing the associations on the basis of the type of intervention and clinical context; (III) organising the results into thematic categories; and (IV) assessing the quality of the studies, methodological limitations, and consistency of the results across different settings. This approach allowed the identification of common themes in nursing practice related to the care of patients with delirium. No statistical effect measures (e.g., risk ratios, mean differences) were calculated, as the heterogeneity of study designs and outcomes precluded quantitative synthesis. Studies were assigned to one of the three synthesis categories (diagnosis, prevention, or therapeutic interventions) on the basis of the primary focus and type of nursing activity described. This categorisation was guided by tabulated study characteristics, including intervention type, setting, and outcome domain. The overall selection process is illustrated in a PRISMA 2020 flow diagram (Figure 1).

### 2.6. Study Risk of Bias Assessment

The assessment of the bias risk of each study was performed independently by two reviewers using predetermined methodological criteria, following the PRISMA guidelines, and contributed to the overall interpretation of the evidence. Factors such as clarity of the study’s purpose, appropriateness of the research design to the questions posed, method of sampling, clarity of results presentation, and presence of potential conflicts of interest were considered. In the case of discrepancies between assessments, decisions were made through discussion or with a third reviewer. We applied the Checklist for Qualitative Research (JBI Critical Appraisal Tools) [32] and the AMSTAR-2 tool [33] to assess the methodological quality of the included studies. The bias risk assessment served as one of the criteria in the final interpretation of the quality and reliability of the collected data.

The potential risk of reporting bias (bias due to missing or selectively reported results) was assessed qualitatively. The reviewers compared the outcomes reported in the included studies with those specified in the study objectives and methods sections. No formal statistical assessment was performed, as the synthesis was descriptive.

## 3. Results

The review yielded a total of 3790 relevant studies. After the titles and abstracts were reviewed, 408 papers were included. After analysis, a total of 39 literature items that met the selection criteria were obtained. The largest groups consisted of systematic reviews and meta-analyses (*n = 13*), and original papers (*n = 13*), followed by systematic reviews (*n = 6*). Other types of articles included meta-analyses (*n = 2*), and individual publications: secondary analyses of survey data, scoping reviews, umbrella reviews, narrative reviews and Cochrane reviews (each *n = 1*).

In our work, certain articles, such as [34], were excluded. The main focus is on patient experiences rather than specific nursing activities or nursing interventions in relation to the assessment, prevention, or treatment of delirium [34]. Articles concerning nursing home residents were excluded [35]. We considered the conditions of patients in hospitals.

### 3.1. Characteristics of the Included Studies

The review included studies that met the inclusion criteria. The studies were conducted in Europe, Asia, North America, and Australia, among other places, which increases their external validity. Table 2 contains the detailed characteristics of the included studies.

On the basis of the numerical summary, most studies focused on patients in ICUs and critically ill patients, with nine articles in each of these groups. Studies on patients hospitalised outside intensive care units or from various hospital wards were equally well represented (*n = 23*). A slightly smaller percentage of studies concerned postoperative and surgical patients (*n = 6*), whereas individual studies covered specific populations, such as perioperative patients (*n = 1*), elderly people (*n = 4*), patients’ families (*n = 1*), and emergency department patients (*n = 1*) (Table 2). In addition, three articles concerned nursing staff, including nurses working in intensive care units, which indicates an interest in the subject of knowledge, attitudes, and professional practices in the care of patients with delirium. The studies analysed focused primarily on critically ill patients hospitalised in ICUs, whereas studies involving other clinical groups were of lesser importance.

In the present review of works, the most numerous group consisted of publications on nonpharmacological interventions (*n = 17*). The review also included articles on multicomponent interventions (*n = 3*), interventions targeting sleep and circadian rhythm (*n = 3*), cognitive function/anxiety/education/staff stress (*n = 3*), and pharmacological and mixed interventions (*n = 2*). A slightly smaller percentage of studies concerned sensory interventions, family participatory interventions, cognitive aspects/staff stress and factor analysis.

Some studies have focused on ICUs, where patient monitoring is continuous and covers critical conditions [33,40,59]. Others focus on general hospital wards, where nursing interventions must be more targeted and adapted to less intensive care settings [43]. Some publications describe comprehensive multicomponent interventions covering assessment, prevention, and treatment [56,58,59]. Others focus mainly on the diagnosis and early detection of delirium symptoms, without discussing therapeutic interventions in detail [39]. Some articles are primary research studies, including RCTs and nurse-led studies [33,58]. Others are systematic reviews or meta-analyses covering multiple studies, which allow for the generalisation of results but limit the detailed description of nursing practices [23,39,40,56,59] (Table 2).

### 3.2. The Role of Nurses in Delirium Diagnosis

An analysis of selected publications indicated that nurses play a key role in the early recognition of delirium, patient monitoring (observation, documentation) and communication with the medical teams. Among the analysed studies, 10 articles referred to the diagnosis of delirium. These studies concerned patients [11,12,18,41,46,60,66] and nursing staff [21,39,57]. The goal of nurses is to recognise and identify delirium early via appropriate tools. Among the publications analysed, particular emphasis on the diagnosis of delirium was placed on work [11,12,18,21,39,41,46,57,60,66]. Goettel and Wueest focused directly on diagnostic methods for delirium [12]. However, it should be noted that most of the studies covered a much broader range of topics and were not devoted exclusively to the diagnosis of delirium (Table 3). For example, Wang and colleagues studied psychological stress in nurses caring for patients with delirium, and this stress affected their ability to assess and recognise the disorder [57]. In turn, Koirala et al. and McCoy et al. provided epidemiological data that serve as background information justifying the need for effective delirium diagnostics [11,18]. Other studies have focused on nurses’ knowledge, attitudes, practices, stress and factors associated with caregiving that affect their ability to correctly diagnose this disorder [21,57].

Despite the common goal of improving the quality of care for patients with delirium, studies differ in terms of the clinical setting [12,18,41,46,60] scope of intervention, and assessment methods used. Most papers emphasise that nurses are the first link in identifying symptoms of delirium, because of to their daily contact with patients [10,21,23,33,35,39,40,56,58,59]. In most studies, nurses use standardised tools such as the CAM-ICU, 4AT, or ICDSC, which allow for systematic monitoring of patients in different hospital wards [39,56,59]. Notably, however, the 4AT and ICDSC are primarily screening tools, whereas the CAM is used for the diagnostic confirmation of delirium. Early identification of delirium symptoms by nurses is emphasised in all analysed studies as crucial for reducing the severity and duration of delirium [23,33,35,58].

Despite differences in clinical settings, methodologies, and scopes of intervention, all studies emphasise the key role of nurses in diagnosing and monitoring delirium. The common conclusion is the need for systematic assessment of patients and the use of standardised diagnostic tools, which enables early intervention and improved clinical outcomes. Differences between studies, however, indicate the need to adapt nursing strategies to the specific characteristics of the ward and the individual needs of patients. The literature shows a lack of interventional studies evaluating the effectiveness of training programs aimed at improving nurses’ diagnostic skills in recognising delirium. Existing studies focus mainly on describing the level of knowledge and attitudes. The impact of occupational stress and workload on the accuracy of delirium diagnosis also remains poorly understood—there are mainly cross-sectional studies, without analyses of cause-and-effect relationships. In addition, research on the diagnosis of delirium in nursing mainly covers intensive care and surgical wards, whereas other clinical contexts, such as emergency, geriatric, and neurological wards, are underrepresented, limiting the possibility of generalising the results to a wider patient population.

### 3.3. Preventive Interventions Undertaken by Nurses

Most publications (*n = 24*) concerned prevention, preventive measures, nonpharmacological interventions, and care programs [13,14,32,33,34,38,39,40,41,43,44,45,46,49,50,51,52,54,55,57,59,60,61] (Table 3). In the studies included (Table 1), the effectiveness of multicomponent nonpharmacological interventions conducted by nurses for preventing delirium varied considerably depending on the clinical setting and study design. While several meta-analyses and original studies have shown beneficial effects, particularly in reducing the incidence of delirium, others have shown no statistically significant improvement in primary outcomes such as duration, severity, or mortality. The positive effects of interventions carried out by nurses have been noted in systematic reviews and original studies focusing on multicomponent strategies combining orientation, sleep hygiene, sensory optimisation, and family involvement [38,39,40,45,46,49,50,52,59,60,61]. These interventions, often implemented by multidisciplinary teams, are associated with a reduction in the duration of delirium, improved patient orientation, or a shorter stay in the intensive care unit. For example, the Dy-Del program effectively reduced both the incidence and duration of delirium [48], whereas multicomponent protocols based on sleep and circadian rhythms showed similar benefits in intensive care unit patients and perioperative patients [45,46,58]. Several studies emphasise that effective delirium prevention is primarily based on nonpharmacological interventions, which are an integral part of a nurse’s work [13,23,39,52]. Publications emphasise the role of nurses in implementing nonpharmacological strategies, such as reorientation, cognitive stimulation, early mobilisation, and family support, which aid in the prevention and treatment of delirium [23,39,40,58,59]. The most commonly reported nonpharmacological strategies include orienting the patient in time and space. Other important measures include ensuring adequate pain control while limiting medications with depressant effects on the nervous system, as well as promoting early mobilisation and cognitive activation [65]. Special emphasis is placed on the implementation of multicomponent prevention programs that integrate several of these interventions simultaneously [44]. Some studies conducted in specific clinical contexts reported no significant reduction in delirium incidence following nurse-led or multicomponent preventive programs, especially in surgical, cardiac-surgery, and geriatric settings [46,49,52,55,60,64].

Nurses play a key role in preventing delirium in hospitalised patients, both in general wards and ICUs. Daily contact with patients enables early detection of the first signs of cognitive impairment and the implementation of preventive measures that minimise the risk of delirium [10,21,23,33,35,39,40,56,58,59]. Delirium prevention is a key element of nursing care in various hospital wards, especially in intensive care and geriatric care. The most commonly used preventive measures include eight main strategies that complement each other and aim to reduce the risk of cognitive impairment. The most commonly used preventive nursing interventions include: (I) systematic monitoring and assessment of patients [32,34,39,40,41,43,49,52,56,59]; (II) cognitive reorientation and stimulation, which include reminding patients of the date, location, and purpose of hospitalisation, as well as brief mental exercises and conversations that support their cognitive function [23,33,40,44,51,58,60]; (III) early mobilisation and physical activity. Early mobilisation, bedside exercises, and mobilising patients as much as possible are important elements of delirium prevention, reducing the risk of complications and improving patients’ overall functional status [14,33,40,44]; (IV) patients’ sense of comfort. Nurses ensure adequate lighting, noise reduction, a bed ensuring patient comfort and proper patient positioning. Activities also include pain control, hydration and nutrition, and elimination of stressors [23,32,39,50,55,57,61]; (V) family support and communication, which has a positive effect on reducing the risk of delirium [23,33,39,54,58,59]; (VI) adapting to the patient’s circadian rhythm. Organising sleep and rest in accordance with the natural circadian rhythm and limiting nighttime disturbances promotes cognitive stability and reduces the risk of delirium [45,46,58]; (VII) patient and healthcare team education. Nurses play an educational role, informing patients and team members about the risk factors for delirium and methods of prevention [13,23,33,35,60]; and (VIII) multicomponent interventions [13,14,34,36,38,40,43,44,49,55,61]. Combining the above measures into comprehensive prevention programs allows for more effective delirium prevention. Examples include the integration of early mobilisation, environmental control, cognitive stimulation, family support, and regular monitoring of the patient’s condition [39,56,59].

However, many high-quality studies and reviews have not confirmed the positive effects of interventions carried out by nurses. Studies [33,41,42,43,44] have shown that despite the active involvement of nursing staff, preventive programs do not lead to a significant reduction in the incidence or duration of delirium. For example, stepped-wedge RCT studies conducted in intensive care and neurology wards [33,42] and systematic reviews conducted by [41,43] did not show statistically significant differences between the intervention and control groups. Furthermore, a review [51] revealed that although multicomponent interventions may help prevent delirium, their effect on duration or severity remains limited. These inconsistent results may reflect differences in interventions, patient populations, and methodological limitations. Many studies have reported small sample sizes, heterogeneous outcome measures, and inconsistent delirium assessment tools [32,41,42,44,46,56].

The management of modifiable risk factors remains a key component of effective delirium prevention. Further multicenter studies, particularly in the areas of intensive care and neurology, are needed to develop effective, integrated strategies for the treatment and prevention of delirium. Despite the growing number of studies, the literature indicates certain gaps. There is a lack of uniform protocols in general wards, data on the long-term effects of prevention in specific populations are limited, and the heterogeneity of studies makes comparisons between different settings difficult [13,40,53]. Taken together, current evidence suggests that nurse-led multicomponent interventions are promising but not universally effective. Their success likely depends on adequate staff training, the patient population, and adherence to standardised protocols.

### 3.4. Therapeutic Interventions Undertaken by Nurses and Delirium Management

Nurses play an important role not only in the prevention of delirium but also in its treatment and management. Their activities include monitoring symptoms, implementing nonpharmacological interventions, supporting pharmacological treatment, and coordinating activities within a multidisciplinary team [10,23,33,39,40,56,58,59]. Nurses take an active part not only in diagnosis and prevention, but also in supportive treatment of patients diagnosed with delirium (Table 3). Their therapeutic interventions include both direct actions and cooperation with an interdisciplinary team [63]. The most commonly used therapeutic interventions include the following: (I) monitoring and early response to symptoms of delirium, enabling rapid implementation of therapeutic measures [23,33,35]; (II) nonpharmacological interventions-cognitive reorientation and stimulation, assisting in maintaining orientation, and conversations supporting cognitive functions; environmental control and patient comfort, adequate lighting, and noise reduction, ensuring a comfortable position, and eliminating stressors; mobilisation and physical activity, bedside exercises or getting up as much as the patient is able; emotional and family support, the involvement of family in care and the provision of a sense of security and emotional support [39,40,58,59]; and (III) supporting pharmacological treatment. Nurses monitor the administration of drugs to treat delirium, including controlling doses, observing adverse effects, and working with physicians to adjust treatment [23,56]; (IV) coordination of a multidisciplinary team. Delirium management requires collaboration among nurses, physicians, physical therapists, psychologists, and patients’ families to implement a comprehensive therapeutic approach and monitor the effectiveness of interventions [33,39,59]; (V) tailoring interventions to individual patient needs. Nurses modify therapeutic measures depending on the patient’s clinical condition, risk of complications, and comorbidities, which increases the effectiveness of treatment and patient safety [35,58].

Research indicates that measures such as early mobilisation [56], effective pain management, proper nutrition, and hydration can lead to up to a 40% reduction in incidence in older adults [13]. A study by Sist and colleagues involving nurses revealed that nursing staff give the highest priority to activities that ensure patient safety, followed by interventions that support communication and continuous supervision [39]. Lower priority was given to activities related to family involvement and modification of the physical environment [39].

Differences between publications on therapeutic nursing interventions can be observed in several aspects. First, they differ in the clinical setting in which the studies were conducted. Some focus on ICUs, where patients are in critical condition and delirium monitoring is continuous and requires a rapid response [33,40,59]. Others concern general hospital wards, where nursing interventions are less intensive and activities are tailored to patients with a lower risk of delirium [23,39]. There are also differences in the scope of interventions. Publications Jang and Lee [51] and Tovar and Castano [52] concern active therapeutic measures taken by nurses aimed at reducing the symptoms of delirium in patients already affected by this disorder, rather than merely its prevention. The authors demonstrated that reducing anxiety levels through nonpharmacological measures (including emotional support, relaxation techniques, and therapeutic communication) translates into improved cognitive function and reduced severity of delirium [51]. They emphasise the importance of the therapeutic aspect of nursing care, which goes beyond traditional nursing activities and includes elements of supportive psychotherapy and behaviour modification [51]. In turn, a previous publication [52] described a complex nursing-led intervention program for critically ill patients. This intervention includes continuous assessment of delirium symptoms, care, emotional support, cognitive orientation, and maintenance of circadian rhythm, which, in a clinical trial, resulted in a significant reduction in the frequency and duration of delirium [52]. Both articles focused on therapeutic measures taken after the onset of delirium. However, they differ in scope [51] and focus on the psychological dimension of therapy, whereas [52] describes a comprehensive, practical model of nursing therapeutic intervention in intensive care.

Some publications describe multicomponent interventions that combine patient monitoring, cognitive stimulation, mobilisation, environmental control, family support, and cooperation with the therapeutic team [39,56,59]. Other articles focus mainly on selected nonpharmacological measures, such as reorientation or emotional support, without considering pharmacotherapy or team collaboration [35,58]. Another aspect is the inclusion of pharmacotherapy. Some articles describe the role of nurses in monitoring and supporting pharmacological treatment, including controlling drug doses and observing adverse effects [23,56]. Others focus exclusively on nonpharmacological interventions and do not analyse aspects of pharmacotherapy in detail [39,40,58]. Differences are also evident in the research methodology. Some works are primary studies, including randomised controlled trials and nurse-led studies, allowing for the assessment of the direct impact of nursing interventions [33,58]. Others are systematic reviews or meta-analyses that synthesise the results of multiple studies, allowing for general conclusions but limiting the description of specific nursing actions in practice [23,39,40,56,59]. The last difference concerns the degree to which interventions are tailored to individual patient needs. Some publications emphasise the importance of individualising interventions, taking into account age, comorbidities, and delirium risk level [35,58]. Others focus mainly on standard protocols and procedures, with less emphasis on personalising interventions [39,59]. All these differences highlight that although nurses play a central role in delirium management, the scope, intensity, and nature of interventions may vary depending on the clinical context, study methodology, and individual patient needs.

Nurses are key participants in delirium management, performing both therapeutic activities and coordinating the care team. Nonpharmacological interventions, early identification of symptoms, and monitoring of pharmacological treatment can reduce the severity of delirium, shorten its duration, and improve patients’ quality of life. Although several studies have confirmed the effectiveness of multicomponent, nurse-led interventions in preventing delirium [39,56,60], several high-quality studies have not reported significant effects [33,41,42,44]. This mixed body of evidence indicates that the impact of such interventions may depend on the clinical context, patient characteristics, and implementation fidelity. Therefore, conclusions about the universal effectiveness of nurse-led interventions should be interpreted with caution.

## 4. Discussion

The role of nurses in caring for adults with delirium in hospitals is multifaceted. It focuses on identifying risk factors, preventive measures, recognising symptoms, and participating in the treatment of patients with delirium.

An analysis of the literature revealed that the role of nurses in caring for patients with delirium varies depending on the type of hospital ward, but all studies emphasised common elements of nursing care, primarily the importance of early recognition of delirium symptoms and systematic monitoring of risk factors [12,23,30,65,67,68,69]. Studies have identified many factors that trigger delirium, including acute clinical conditions (e.g., sepsis, stroke, severe liver failure, advanced heart failure, and infections); injuries (including head injuries, fractures, and multiple organ injuries); dehydration, constipation, uncontrolled diabetes, and immobilisation; the effects of certain medications (benzodiazepines, dihydropyridines, antihistamines, anticholinergics, and opioids); and surgical interventions, especially in patients with comorbidities [6,70]. In the context of postoperative delirium, the authors emphasise that its occurrence results from the interaction of factors related to the patient, the course of the surgical procedure, and the anaesthesia used [25]. Symptoms usually appear within the first five days after surgery, with the most severe symptoms occurring between the first and third days [12,71]. Risk factors for delirium can be categorised as non-modifiable or modifiable. Non-modifiable factors include advanced age, visual and hearing impairments, a history of stroke, dementia, and previous episodes of mental disorders. Modifiable factors include hypertension, dehydration, metabolic disorders, anaemia, and inadequate pain management [3,5,6,56].

The results of the review confirm that the assessment of the risk of delirium should consider the patient’s clinical condition, procedural factors (e.g., type of surgery, method of anaesthesia), and environmental factors (e.g., isolation, confusion about time and place) [59,69]. Notably, delirium has a significant negative effect on the health of patients, especially in the elderly population. The most common consequences include prolonged artificial ventilation, increased risk of complications and adverse events (e.g., falls), cognitive impairment (e.g., memory loss, development of dementia), prolonged hospitalisation, increased treatment costs and mortality [5,6,7,16,37,56,72,73,74]. For this reason, preventive and observational measures taken by nurses, consisting of systematic monitoring of patients and early recognition of risk factors and symptoms of delirium, play a key role [3,12]. The effective methods for preventing delirium vary depending on various factors [13]. The differences in the effectiveness of interventions across individual studies may be partly due to organisational factors. Differences in nursing staff workload, employment rates, and level of training in delirium may have a significant effect on the effectiveness and success of prevention programs. Several studies have identified limited staff availability and inadequate training as barriers to the consistent implementation of nonpharmacological interventions [37,45,53]. These findings suggest that the success of delirium prevention strategies depends not only on the intervention design itself, but also on the work environment and institutional support. Nurses, as individuals who are in close and constant contact with patients, should be knowledgeable about the main risk factors for delirium and skilled in identifying it in their daily clinical practice. Scientific evidence confirms that effective monitoring of delirium contributes to reduced mortality and a shorter duration of mechanical ventilation, especially in surgical patients requiring intensive care [44,75]. The diagnostic process should involve conducting an interview, reviewing the medical history, and assessing the patient’s current clinical condition [3]. It is particularly important to compare the patient’s current mental state with their baseline state, as this allows for the identification of subtle changes in behaviour that may indicate the development of delirium [12,19]. The observations of family members and caregivers can also be valuable sources of information, allowing sudden cognitive changes and their fluctuations to be identified [76]. Particular attention should be given to attention disorders (e.g., difficulty concentrating) and changes in consciousness (e.g., agitation or apathy). The results are equally inconclusive with respect to the impact on the duration of delirium [44]. The most commonly described measures in the literature focus on continuous documentation of symptoms and their variability, as well as individualising care according to the type of delirium [44].

One of the significant clinical challenges remains distinguishing delirium from dementia, especially in older people [5,76]. Delirium is characterised by sudden disturbances in attention and changes in consciousness (confusion, difficulty perceiving the environment) and impairments in cognitive functions such as memory, speech, and thinking. In dementia, however, symptoms develop gradually, and consciousness and attention remain normal [76]. In qualitative studies, nurses reported difficulties in distinguishing delirium from dementia, as well as uncertainty in the use of available diagnostic tools [21,26]. Standardised screening tools such as the CAM, CAM-ICU, 4AT, ICDSC, and Nu-DESC are essential for the rapid identification of patients at risk of delirium [12,41,77]. Research has shown that the use of screening tools varies depending on the country and type of department [78]. The ICU-CAM and ICDSC dominate in ICUs, and the 4ATs in rehabilitation units, whereas clinical assessment without tools is often used in emergency departments and nursing homes. Unfortunately, many studies indicate the low use of screening tools in clinical practice, resulting, among other things, from a lack of training, work overload, and insufficient systemic support [21,60,79,80]. According to current international guidelines (updated in 2025 and published by the Society of Critical Care Medicine in 2018), all hospitalised patients should be routinely assessed for the risk of delirium (National Institute for Health and Care Excellence-NICE recommendations) [12,24,81]. A correlation was observed between nursing staff participation in training and the frequency of use of standard diagnostic tools [78]. Studies have shown that as nurses’ knowledge and awareness of delirium increases, their ability to assess and manage delirium improves significantly [82].

Depending on the level of psychomotor activity, delirium can be divided into three subtypes: hypoactive, hyperactive, and mixed [3,83,84]. In hospital settings, the hypoactive subtype is most commonly observed, and is characterised by reduced reactivity [11,83]. Patients are often apathetic, drowsy, and withdrawn, with a limited attention span [13]. In addition to those in the ICU, reduced mobility, slowed reactions, impaired concentration, and decreased appetite are also observed [81]. The hypoactive subtype of delirium is more persistent than the hyperactive subtype [85] and is often associated with poorer long-term cognitive functioning [7,74,85]. The patient is overly alert, highly agitated, restless, uncooperative, emotionally unstable, and exhibits hyperactive delirium. Hyperactive delirium is easier for medical personnel to recognise [2,19]. Common features are found in the mixed subtype. It is estimated that the hypoactive subtype accounts for 11–17% of people treated in the ICU, the mixed form accounts for 7–10%, and the hyperactive form accounts for 4% [83,84]. However, evidence suggests that nurses have low knowledge about delirium and difficulty recognising the hypoactive subtype in particular [21,86]. It is generally more difficult to diagnose, as evidenced by the limited number of studies in the scientific databases searched. Nurses do not routinely use validated tools to diagnose delirium in their work. This may be due to workload, reluctance, or ignorance [21,86]. Nurses’ attitudes towards patients with delirium are influenced by their experience, seniority, age, and education, and participation in training correlated with knowledge and practice in the care of patients with delirium. Therefore, it is essential for all nurses, regardless of their age and experience, to deepen and update their knowledge. Nurses find it difficult to identify modifiable risk factors that should be targeted by nonpharmacological interventions, especially in the prevention of delirium. Current recommendations based on the guidelines of the Society of Critical Care Medicine suggest multicomponent nonpharmacological strategies for the prevention or treatment of delirium [87]. Nurses should implement nonpharmacological interventions, but evidence suggests that this is not the case, and that the incidence of delirium remains unacceptably high [28]. Numerous interventions are available in everyday clinical practice, but only a few have a high or moderate level of reliability [36,55]. For example, cognitive function can be stimulated by recalling personal memories, whereas patient orientation is maintained through regular communication [88], conversation, and the involvement of a familiar family voice. Ensuring adequate hydration and nutrition is essential [89], whether by encouraging oral fluid and food intake or, when necessary, administering intravenous therapy. Providing sensory aids such as glasses and hearing aids, fitting dentures, and educating family members about delirium symptoms and supportive strategies are equally important [90]. Additional interventions involve assessing and optimising oxygenation [42,65], early identification and treatment of infections, prevention of postoperative complications through monitoring and adherence to infection control guidelines, and prevention of falls and pressure ulcers. Creating a supportive hospital environment that is well lit during the day and quiet and darkened at night and incorporating music interventions as a form of supportive therapy have also been reported as effective strategies [50]. Reported strategies also include the use of music therapy as a form of supportive care and the avoidance of unnecessary catheterisation [65,66,91]. Mobilisation is encouraged, including early postoperative ambulation, with appropriate walking aids provided if needed, as well as active range of motion exercises even for immobile patients [13,44,65]. Pain assessment is emphasised, particularly in patients with communication difficulties, such as those with tracheostomy, ventilator support, or dementia. Light sedation is preferred over deep anaesthesia, which may help reduce the incidence of postoperative delirium [13]. Effective delirium management also relies on collaboration within the therapeutic team [44], involving participation in clinical decision-making and monitoring of treatment effects [44,58,67]. Family members are actively involved in care and decision-making processes, and they are educated about delirium symptoms and methods to support their loved ones [7,13,37,42,44,52,56,74,81,87]. While nonpharmacological interventions have been shown to be effective in populations hospitalised outside the ICUs, they have not shown a consistent effect on treatment in the critical care setting [92]. Research does not clearly show that the use of nonpharmacological preventive strategies implemented by nurses in ICUs to reduce the frequency and duration of delirium is effective [14,37,45,47,89,93].

Examples of comprehensive intervention packages include (I) the SP-CS-EM-PC-AS program (Sleep Promotion–Cognitive Stimulation–Early Mobilisation–Pain Control–Assessment Strategy). This intervention package, proposed by Matsuura and colleagues [63], among others, has been shown to be effective in reducing the incidence of delirium. The greatest benefits were observed with the simultaneous use of cognitive stimulation and sleep promotion; (II) The ABCDEF package, recommended by the Society of Critical Care Medicine as part of the ICU PADIS guidelines [24], encompasses several key components: assessment and treatment of pain (A), spontaneous breaks in ventilation and sedation (B), selection of appropriate sedatives (C), daily assessment of delirium (D), early mobilisation and exercise (E), and family involvement (F). The implementation of this bundle has been shown to effectively reduce the incidence of delirium, the duration of mechanical ventilation, and the length of hospitalisation, although practical challenges may limit its application; (III) the Hospital Elder Life Program (HELP), designed for nonintensive care units, focuses on six key strategies: improving sleep, providing sensory support, promoting mobilisation, ensuring nutritional support, maintaining reality orientation, and encouraging social-emotional engagement [42]. This program is widely implemented in the USA and Europe and has been shown to effectively reduce the incidence of delirium in patients aged 65 years and older who are hospitalised for surgical or medical reasons [94]. Protocols aimed at preventing delirium should prioritise the optimisation of modifiable risk factors [2]. According to NICE recommendations, patients identified as being at risk of developing delirium should receive care from familiar healthcare personnel, while all preventive interventions should be implemented by an interdisciplinary team specifically trained in delirium prevention and management [81].

Despite the availability of effective protocols for preventing delirium, their implementation in clinical practice often fails due to various factors, including heavy workloads for nursing staff, insufficient training, lack of standard procedures, poor communication within the interdisciplinary team, limited institutional support, insufficient knowledge of diagnostic tools (CAM, 4AT, etc.) or their improper use, and lack of regular training. Overcoming these barriers is crucial for translating evidence-based strategies into real improvements in patient outcomes.

In summary, current evidence does not provide a clear answer for the selection of optimal nonpharmacological interventions, so it is very difficult to create uniform procedures to standardise the approach for the prevention and treatment of delirium. Moreover, no studies to date have identified interventions in relation to different types of delirium, which remains an important research gap. The focus on delirium in hospitals is justified because the impact of delirium is not limited to the patient alone, but also affects their environment, including their family. It is, therefore, the task of nurses to educate caregivers about what delirium is and how to provide care when delirium occurs.

## 5. Limitations

Although the literature review provides valuable information, it is not without certain limitations regarding the scope of the analysed works, the availability of the latest data, and the risk of publication bias, which may affect the completeness and generalisability of the conclusions. The most important of these are as follows: (I) most of the included works are review articles; (II) despite the consistency of the analysed publications the nurses’ working conditions vary significantly across countries; and (III) although the review was prepared in a systematic manner, there is a risk that some relevant material may have been omitted owing to limited access. An additional difficulty was the limitation of the analysis to publications in English and Polish only, which may have resulted in other valuable studies not being taken into account.

## 6. Implications for Practice

The evidence from multiple studies highlights the central and multifaceted role of nurses in preventing and managing delirium in hospitalised patients, particularly in intensive care and postoperative settings. Nurses are uniquely positioned to implement both nonpharmacological and pharmacological interventions because of their continuous patient contact and holistic understanding of patient needs. They are responsible for early detection, risk assessment, and timely initiation of interventions aimed at minimising the incidence, duration, and severity of delirium. A key implication for practice is the need for the systematic use of standardised screening and diagnostic tools, such as the CAM-ICU and 4AT, by nursing staff. Early recognition of delirium allows prompt intervention, reducing complications such as long-term cognitive impairment or prolonged hospital stays. Nurses’ daily engagement with patients places them at the forefront of monitoring subtle behavioural changes, ensuring early identification and management of delirium. Multicomponent, nonpharmacological interventions, such as reorientation, cognitive stimulation, sleep hygiene optimisation, sensory interventions, and mobilisation, require active nursing involvement for effective implementation. Nurses not only deliver these interventions but also coordinate family engagement, enhance environmental conditions, and support individualised patient care. Evidence shows that programs such as M.O.R.E. and Dy-Del, led by nursing teams, can significantly reduce delirium incidence and improve patient quality of life [51,52]. However, translating this evidence into routine clinical practice remains challenging. Nursing workload, staff shortages, and variations in knowledge or experience can limit the consistent application of delirium prevention protocols. The implementation of multicomponent interventions may also be constrained by limited time, inadequate staffing levels, and the absence of institutional policies that prioritise delirium prevention. Therefore, while the role of nurses is central, the feasibility of sustaining these interventions depends on organisational support, including adequate nurse-to-patient ratios, access to training programs, and interdisciplinary collaboration. Targeted education and training, including skills in hypoactive delirium recognition, sensory interventions, and circadian rhythm-based care, are vital to enhancing nurses’ competence and confidence. Moreover, psychological support and resilience-building initiatives for nurses are also important, as caring for patients with delirium can be stressful and emotionally demanding. Ultimately, the success of nursing-led delirium prevention strategies requires not only individual skills but also a systemic commitment to creating work environments that enable consistent, high-quality care.

## 7. Conclusions

Multicomponent, nurse-led interventions show promise in preventing and managing delirium, particularly in surgical and geriatric settings; however, evidence from ICU populations remains inconclusive. Early and systematic identification of delirium not only enables timely clinical intervention but also facilitates the tailoring of care to the individual patient’s needs, such as cognitive stimulation, orientation strategies, environmental modifications, and optimisation of hydration and nutrition. Interdisciplinary collaboration ensures that all members of the healthcare team contribute their expertise to a coordinated care plan. Furthermore, structured training programs and standardised care protocols for healthcare staff are essential for maintaining consistency in delirium management across hospital settings. Such initiatives enhance staff knowledge and competence, promote adherence to evidence-based practices, and foster an organisational culture that prioritises early recognition, prevention, and comprehensive management of delirium.

In summary, future research should aim to clarify which components are most effective in specific clinical contexts and how implementation fidelity influences outcomes.

## Figures and Tables

**Table 1:** The selection of articles included in the analysis.

	Number	Free Full Text	By Titles	By Abstracts	Included	Duplicates
PubMed/MEDLINE	1356	674	172	40	20	
Scopus	1310	686	118	46	12	
CINAHL/EBSCO	1125	542	118	55	8	
Total:	3791	1902	408	141	40	1291

**Table 2:** Detailed characteristics of included studies.

Number	Reference	Setting/Population/Country	Publication Type	Scope of the Study	Main Conclusion	The Main Conclusions Regarding the Role of Nurses	Study Bias
1.	[36]	Patients hospitalised out of ICU	Systematic review (22 studies included)	Nonpharmacological interventions	Complex, nonpharmacological interventions have little or no effect on patient mortality in the hospital. However, they can shorten the duration of delirium episodes and reduce the length of hospitalisation.	Nurses play a role in implementing multicomponent interventions to prevent delirium, particularly through protocols such as reorientation, cognitive stimulation, and sleep hygiene. They are involved in assessing and addressing delirium risk factors, indicating a proactive role in prevention.	Multicomponent nonpharmacological interventions show limited effectiveness. They can reduce delirium incidence and duration, but do not significantly impact patient mortality. Study interpretations are limited by: lack of blinding (risk of bias), scarce data on patients with dementia, limited evidence for single-component interventions, inconsistent frailty assessment and subgroup reporting, insufficient attention to cognitive outcomes and dementia progression.
2.	[37]	ICU patients (N = 2566)/Australia	Original study	Multicomponent interventions carried out by nurses	Overall, the nursing-led intervention did not produce a meaningful decrease in either the occurrence or the duration of delirium.	Nurses play a role in delivering and promoting the delirium prevention protocol. However, despite their involvement, the intervention did not lead to a statistically significant reduction in either the incidence or the duration of delirium and, thus, failed to achieve the expected outcomes.	Owing to the lower-than-anticipated baseline incidence of delirium (14%), the achieved sample size lacked sufficient statistical power to detect the observed effect size.
3.	[12]	Perioperative and ICU patients	Review (narrative review)	Diagnostic approaches to delirium	Timely diagnosis and treatment of delirium are critical to preventing serious adverse outcomes such as death and institutionalisation. More than 30 tools are available for screening and diagnosing delirium, but they vary in sensitivity, specificity, and time to diagnosis, which complicates their selection and comparison.	Nurses, due to their daily patient contact, can detect early signs using reliable screening tools (e.g., CAM-ICU, 4AT). Timely diagnosis prevents complications, and regular use of screening tools is essential for effective nursing care and improved patient outcomes.	An excessive number (more than 30) of diagnostic tools complicates selection and comparison in studies, thereby complicating the process of standardisation and comparison of diagnostic methods. Healthcare professionals may not be sufficiently familiar with the various assessments of delirium, indicating a gap in education and training.
4.	[38]	Patients hospitalized out of ICU	Systematic review and meta-analysis (39 studies included)	Preventive, multicomponent interventions	Multicomponent interventions significantly reduce the incidence of delirium in hospitalised patients compared to usual care. The study shows that monitoring the depth of anaesthesia using Bispectral Index-guided anaesthesia reduces the incidence of postoperative delirium compared to anaesthesia without such monitoring.	Nurses play a central role in multidisciplinary teams by participating in educational programs, implementing protocols that address specific risk factors, and delivering specialised interventions such as medication management and mobilisation.	Evidence for delirium prevention outside the ICU is limited, except for multicomponent interventions. Many studies had small samples, high heterogeneity, and lacked data on key subgroups (e.g., patients with dementia). Most did not exclude patients with preexisting delirium, affecting accuracy. Outcomes like delirium duration, severity, mortality, functional status, adverse events, quality of life, caregiver or staff burden, and costs were rarely assessed.
5.	[14]	Elderly patients after elective surgery (N = 1470)/Germany	Original study	Preventive interventions	Nonpharmacological multimodal intervention is effective in reducing the risk of delirium in elderly patients undergoing noncardiac surgery.	Nurses played a role in the delirium prevention by participating in training on delirium detection and prevention, implementing intervention modules, evaluating outcomes, mentoring volunteers, and ensuring program feasibility and fidelity.	The intervention did not influence the incidence of delirium in patients undergoing cardiac surgery. Its feasibility and effectiveness should be further evaluated in smaller, nonacademic, and rural hospitals. Patient readmissions after discharge were not reported, and analyses of ICU stays must be interpreted with caution due to variability in institutional protocols.
6.	[39]	Nurses–managing patients with delirium	Systematic review (12 included studies)	Analysis of nurses’ priorities	Nurses generally prioritise patient safety, communication, and monitoring, but individual approaches vary: some tailor interventions to patient needs, others focus on prevention. These differences can delay active delirium treatment and highlight the need for consistent communication, cognitive reorientation, and harmonised decision-making to improve care quality.	Nurses prioritise interventions in delirium management, with a particular focus on safety and communication. Priority patterns vary across levels, reflecting different approaches to care.	The Q-sample was derived from the literature and may have overemphasised certain interventions. The systematic review excluded primary studies included within other systematic reviews. The scenario used to prompt reasoning may have been overly limited, and online data collection could have restricted in-depth engagement. Nurses’ knowledge and prior experience with delirium management were not assessed. Additionally, data collection took place during the pandemic, which may have influenced prioritisation.
7.	[40]	Patients in critical condition	Systematic review and meta-analysis (38 included studies)	Pharmacological and nonpharmacological interventions	Nonpharmacological interventions have not demonstrated a significant impact on delirium prevention or ICU length of stay.	Nurses play a role in preventing delirium in critically ill patients by monitoring sedation, applying sedation protocols, observing patients, and documenting symptoms. Their cooperation in implementing evidence-based pharmacological and nonpharmacological interventions is important.	Many of the included studies did not use blinding, which increases the risk of bias. Data on patients with dementia were limited, and the effectiveness of single-component interventions was not sufficiently documented. Some studies did not exclude patients with existing delirium, which may have affected the assessment of the effectiveness of the interventions. In addition, cognitive outcomes and dementia progression were rarely assessed, and the implementation of multicomponent interventions requires further study.
8.	[41]	Patients from various hospital wards (N = 3257)/United Kingdom	Secondary analysis of survey data	Identifying barriers in the assessment and care of patients with delirium	Delirium management includes nonpharmacological interventions like pain control, hydration, and family visits. Insufficient staffing and training are major barriers to effective care.	Nurses are responsible for assessing delirium and participate in the implementation of nonpharmacological and pharmacological interventions. The barriers reported include staff shortages and lack of time for staff education and training.	All survey data was provided by clinicians participating in World Delirium Awareness Day 2023, without verification of the data provided.
9.	[42]	General wards patients	Systematic review and meta-analysis (17 included studies)	Nonpharmacological interventions	Multicomponent nonpharmacological interventions are effective in reducing the incidence of delirium in general wards. These interventions are effective in both medical and surgical wards.	Nurses implement nonpharmacological interventions to prevent delirium.	Many studies did not report the frequency or duration of nonpharmacological interventions, which contributed to variability in intervention components across studies and made it difficult to standardise multicomponent interventions. This variability, along with the limited number of studies on surgical patients, prevented meta-analysis of single-component interventions.
10.	[43]	Adults admitted to hospital	Systematic review and meta-analysis (9 included studies)	Intervention carried out by nurses	Nonpharmacological nursing interventions for delirium prevention and treatment include multi-component interventions, multidisciplinary care, multimedia education, listening to music, and others. These interventions are effective in preventing delirium, especially in general wards and through multicomponent programs. Interventions are commonly used in surgical wards and are often repeated, with cognitive interventions being a significant component.	Nurses are key in preventing and managing delirium through nonpharmacological interventions, leading teams, implementing strategies independently, supporting families, and contributing to research on improving care.	The interventions were highly heterogeneous due to differences in activities, providers, and timing, and the literature search was insufficient. Only four of the nine studies were assessed as having a low overall risk of bias. Variability in delirium screening scales further complicated comparisons, and the analysis of nonpharmacological interventions delivered by nurses was limited.
11.	[44]	Adults admitted to the ICU	Systematic review (14 included studies)	Nonpharmacological interventions	Nonpharmacological nursing interventions are effective in preventing and reducing the duration of delirium in ICU patients. Multicomponent interventions are the most promising strategy for preventing delirium. Family involvement and cognitive exercises are beneficial in reducing the incidence and duration of delirium.	Nurses play a role in implementing nonpharmacological interventions to prevent delirium in ICU patients. They should involve family members in the prevention process, conduct delirium assessments, and work as part of a multidisciplinary team to minimise risk and prevent delirium.	While multifactorial interventions appear effective, the most beneficial combinations of specific interventions remain unclear, highlighting the need for research that focuses on integrating single interventions into multifactorial approaches.
12.	[23]	Patients in critical condition	Scoping review (15 included studies)	Intervention carried out by nurses	Nonpharmacological interventions-such as sleep promotion, reorientation, cognitive stimulation, early mobilisation, comfort measures, and anxiety reduction-are essential for delirium prevention and management. Early recognition and timely intervention improve outcomes and quality of life.	Nurses are central in assessing, preventing, and treating delirium through both pharmacological and nonpharmacological interventions. In the ICU, they monitor patients, collaborate with medical teams, administer medications, implement multicomponent strategies, and follow guidelines like the ABCDEF bundle, requiring proper training to ensure effective care.	Restrictions in language and free access to full texts may have led to the exclusion of relevant articles. The included studies were heterogeneous and lacked assessment of methodological quality and risk of bias, limiting the strength of clinical recommendations.
13.	[45]	Patients in critical condition	Systematic review and meta-analysis (15 included studies)	Nonpharmacological interventions	Current evidence does not support the effectiveness of nonpharmacological interventions (early mobilisation, cognitive stimulation, sleep promotion, environmental modifications) in reducing the incidence and duration of delirium in critically ill patients.	Nurses play a key role in implementing nonpharmacological interventions by identifying early signs of delirium, and their active involvement and education are essential to the success of these interventions.	The overall evidence quality was low due to high risk of bias, small sample sizes, and heterogeneous interventions, populations, and outcomes. Delirium reporting was inconsistent, many patients were unassessable, and high-risk populations were overrepresented.
14.	[46]	Neurological ICU patients (N = 120)/Netherlands	Original paper	Nonpharmacological interventions	A multicomponent nonpharmacological nursing intervention program did not significantly change the number of delirium-free and noncomatose days after 28 days in patients with neurological ICU admission. The intervention had no significant effect on secondary outcomes such as incidence and duration of delirium.	Nurses play a role in implementing a multicomponent nonpharmacological intervention program aimed at reducing delirium in patients in the neurological ICU. They are responsible for optimising vision, hearing, orientation, sleep, cognitive function, and mobility, the impairment of which is a risk factor, and were able to tailor the program to the individual needs of patients. However, the program did not significantly improve the number of delirium-free and coma-free days after 28 days.	The overall quality of evidence was low due to a high risk of bias. Heterogeneity in interventions, populations, and outcomes prevented data pooling, and some studies had small sample sizes. Inconsistent reporting of delirium incidence and duration, combined with the inability to assess patients in drug- or trauma-induced coma, limited generalisability. The focus on high-risk patients restricted applicability to lower-risk populations, and delirium assessment using CAM-ICU rather than DSM-5 criteria may have underestimated prevalence.
15.	[11]	Adults admitted to hospital	Systematic review and meta-analysis (9 included studies)	Research on the prevalence of delirium	The relationship between immobility, sleep deprivation, and delirium are important issues to consider, especially in intensive care units.	Recommendations for caregivers, including nurses, regarding early diagnosis and treatment of delirium have been confirmed. Nurses should have appropriate tools to monitor delirium in order to recognise it in a timely manner, and intervention recommendations should be widely used. Delirium can be superimposed on dementia.	The studies were heterogeneous in populations and assessment tools, highlighting the need for standardised screening in hospitals. Only English-language and hospital-based studies were included, limiting generalisability. Potential publication bias and methodological diversity restrict the strength of recommendations for guidelines and care models.
16.	[47]	Adults admitted to the ICU	Cochrane review (12 included studies)	Interventions to prevent delirium in the ICU	There is probably little or no difference between haloperidol and placebo in preventing ICU delirium, but further studies are needed to increase confidence in these results. There is insufficient evidence to determine the effect of physical and cognitive interventions on delirium. The effect of other pharmacological interventions, sedation, environment, and nursing care on delirium is unclear and requires further research.	There is potential for nursing interventions to reduce the incidence of delirium in the intensive care unit by targeting predisposing and triggering factors, but more research is needed to confirm their effectiveness.	The quality of evidence is low due to small sample sizes and lack of blinding, highlighting the need for further research to clarify the effectiveness of these interventions.
17.	[48]	Patients after open heart surgery (N = 48)/Iran	Original paper	Multicomponent interventions: preoperative patient education, nursing education, environmental interventions	The intervention did not have a significant impact on preventing or reducing delirium, as differences in incidence, severity, and duration compared to the control group were not statistically significant.	Patient education and nurse training as part of multicomponent interventions did not lead to a statistically significant reduction in the incidence, severity, or duration of delirium following open-heart surgery.	Assessing delirium symptoms only three times a day may overlook fluctuations in severity, which may affect the accuracy of the results.
18.	[49]	Patients in critical condition, ICU patients	Systematic review (118 included studies) and meta-analysis (100 included studies)	Sleep-related interventions	Nonpharmacological interventions significantly improved subjective sleep quality and reduced the incidence and duration of delirium in ICU patients. Specific interventions such as aromatherapy, music, and massage improved sleep, while exercise, family involvement, and others reduced delirium. Light/noise blocking was effective in both improving sleep and preventing delirium.	Nurses should use nonpharmacological interventions that promote environmental compatibility in their clinical practice to improve sleep and prevent delirium in ICU patients.	Excluding studies not published in English or lacking full-text availability is a limitation, as it may have led to the omission of relevant research.
19.	[50]	Postoperative ICU patients	Systematic review and meta-analysis (17 included studies)	Sleep-related interventions	The most effective way to prevent delirium in postoperative patients in intensive care units is through multicomponent nonpharmacological interventions that improve sleep quality by maintaining the circadian sleep/wake rhythm and alleviating stress.	Nurses should pay attention to the patient’s circadian rhythm and stress levels and implement targeted interventions into routine care. The implementation of multicomponent interventions into nurses’ work may generate an increase in workload, which suggests a significant role in the management of these interventions.	Several studies show a risk of bias, indirectness, imprecision, and heterogeneity, leading to very low quality of evidence.
20.	[51]	Neurocritical patients, ICU patients (N = 60)/South Korea	Original paper	Interventions focused on anxiety and cognitive function	Nursing intervention focused on anxiety significantly reduced anxiety levels in the experimental group compared to the control group. The intervention improved cognitive function in the experimental group.	Nurses play a vital role in implementing comprehensive nursing interventions to address psychological and cognitive needs, reduce anxiety, improve cognitive function, and reduce the risk of delirium in patients in neurocritical care units. Nurses’ training is essential because it is critical to the effective implementation and improvement of patient outcomes.	Participants were recruited from a single ICU, limiting the generalizability of the findings. The study assessed only short-term outcomes, and long-term effects on anxiety, cognitive function, and delirium remain unknown. The nonrandomized, controlled design may have introduced selection and measurement bias. Additionally, interventions were implemented under controlled conditions, which may not fully represent real-world practice.
21.	[52]	Patients in critical condition, ICU patients (N = 213)/Colombia	Original paper	Intervention carried out by nurses	The Dy-Del intervention, a nonpharmacological approach involving family engagement, appears effective in reducing both the incidence and duration of delirium, as well as pain intensity, in adult ICU patients.	Nurses play a key role in implementing Dy-Del interventions, which do not involve administering medication but focus on meeting physiological, psychological, spiritual, and social needs. Nurses are crucial in reducing the incidence and duration of delirium and can provide humane care through the use of Dy-Del.	The study did not assess anxiety and stress levels before and after implementing the protocol. Contamination bias may have occurred, as some ICU staff applied elements of the Dy-Del intervention to control group patients. Additionally, the impact of additional workload and adherence to the intervention was not measured.
22.	[53]	Patients in critical condition	Systematic review and meta-analysis (26 included studies)	Nonpharmacological interventions	Family involvement is one of the most effective interventions for reducing the incidence of delirium in critically ill patients. Multicomponent strategies are generally optimal intervention techniques for preventing delirium and reducing the length of stay in the ICU.	The various interventions studied-physical environment intervention (PEI), sedation reduction (SR), family participation (FP), exercise program (EP), improvement of cerebral hemodynamics (CHI), multicomponent training (MLT), and usual care (UC)-highlight the critical role of nurses in engaging families in patient care to help reduce the incidence of delirium.	The study lacks information on the frequency, duration, and number of interventions. Although multicomponent strategies are considered optimal, specific details on how interventions were combined, their frequency, or duration are not provided. Similarly, no detailed information is available for other interventions, including PEI, SR, EP, and CHI.
23.	[54]	Adults admitted to the ICU	Systematic review (17 included studies)	Nonpharmacological interventions	The implementation of a nonpharmacological protocol effectively prevents delirium by controlling risk factors, significantly reducing both its incidence and duration.	Nurse education was an integral part of the protocol, enabling nurses to actively implement nonpharmacological interventions. They play a central role in the M.O.R.E. interventions-Music, Opening of blinds, Reorientation and cognitive stimulation, and Eye/Ear protocols-which led to a significant reduction in delirium in the ICU and improved patients’ quality of life. This highlights the critical role of nurses in successfully delivering nonpharmacological strategies.	The review did not provide a detailed assessment of study bias. Heterogeneity in patient populations, interventions, and outcome measures, as well as the use of different delirium assessment tools, limits comparability and generalisability of the findings.
24.	[55]	Elderly patients	Systematic review and meta-analysis (24 included studies)	Nonpharmacological interventions	Multicomponent interventions effectively prevent delirium. Single-component interventions may help prevent delirium but have limited impact on its duration and severity. Overall, nonpharmacological treatments have limited effectiveness in delirium management.	The use of nonpharmacological interventions to help reduce the incidence of delirium.	The variability in results and quality across systematic reviews makes it difficult to draw definitive conclusions.
25.	[21]	ICU nurses (N = 2835)/China	Original paper	KAP protocol (knowledge, attitudes, practices) concerning hypoactive delirium	A significant percentage of ICU nurses have a positive attitude and adequate practice toward hypoactive delirium, but their level of knowledge is very low. Factors such as age, years of working in the ICU, education, and training in hypoactive delirium are significantly associated with KAP status. Hypoactive delirium is often ignored by nurses and there is no specific nursing procedure that requires improvement in training and standard procedures.	Nurses have poor knowledge of hypoactive delirium. Enhanced training is essential to improve knowledge, attitudes, and practice.	Hospitals were not randomly selected but were chosen through convenience sampling, which may introduce selection bias.
26.	[13]	Patients from different wards	Umbrella review (59 included studies)	Preventive and therapeutic interventions	Delirium prevention and treatment are not well understood, with limited high-certainty evidence. Dexmedetomidine is effective for surgical patients, while nonpharmacological strategies—light sedation, comprehensive geriatric assessment, and multi-component interventions—show benefits, especially in older adults.	The use of nonpharmacological interventions in ICUs and other medical units seems to be confirmed; nurses should use standardised protocols containing multi-component interventions addressing six risk factors: cognitive impairment, sleep deprivation, immobility, visual impairment, hearing impairment, and dehydration. Nonpharmacological interventions may be preferred as first-line treatment.	The review included only meta-analyses of RCTs in hospitalised patients, with most evidence derived from surgical populations and no consideration of delirium subtypes, limiting generalisability.
27.	[56]	Patients in critical condition	Systematic review and meta-analysis (29 included studies)	Nonpharmacological interventions	The most effective nonpharmacological ICU interventions are multicomponent measures-especially early mobilisation and family involvement-which reduce delirium incidence and duration, supporting evidence-based care optimisation for critically ill patients.	Nurses providing care to patients in the ICU can effectively influence the incidence and duration of delirium by implementing several nonpharmacological measures.	The included studies were heterogeneous and of low to moderate quality.
28.	[57]	ICU nurses (N = 355)/China	Original paper	Assessment of psychological stress in ICU nurses caring for delirium patients	Intensive care nurses experience moderate stress caused by caring for patients with delirium. Factors that increase stress include: current experiences related to caring for delirious patients, less knowledge, low satisfaction with support (e.g., organisational), lower psychological resilience of nurses, and low professional self-esteem. There is a need to support nurses who influence these factors.	Caring for patients with delirium can be challenging and stressful for nurses, who often experience moderate levels of work-related stress. Psychological assessment and support are important to safeguard nurses’ mental health and enhance the quality of care. Nurse managers and researchers should prioritise the well-being of ICU nurses by offering targeted support, increasing delirium-related knowledge, and strengthening resilience and coping skills.	The cross-sectional design limits the ability to determine causal relationships. Conducted in only three hospitals in China, and the sample may not be representative of all nurses.
29.	[18]	Patients from different wards (N = 831,348)/United States	Original paper	Characteristics and prediction of delirium frequency	Delirium incidence varies by diagnosis and ward type, with maternity wards showing the lowest rates. Diagnosis and hospitalisation location are stronger predictors than age, highlighting the need for targeted prevention and treatment strategies.	Nurses should take into account the increased risk of delirium in patients hospitalised in high-risk wards.	The present study reported experience at two centres, both of which were tertiary referral centers in the same geographic area. The sample may not be representative of all nurses.
30.	[58]	Family of patients admitted to the ICU	Systematic review (14 included studies)	Family participatory interventions	Single-component interventions, such as familiar voice messages and flexible visits, have shown beneficial effects in reducing delirium. Multicomponent interventions, including family visits with professional support programs and sensory stimulation, have also shown positive effects in reducing the incidence and duration of delirium.	Although it does not directly discuss the role of nurses, those who are closest to the patient can encourage and engage the family during the provision of care.	The results were highly heterogeneous, preventing meaningful data synthesis and limiting the ability to draw definitive conclusions.
31.	[59]	Postoperative patients (abdomen)	Systematic review and meta-analysis (16 included studies)	Interventions to prevent delirium after intra-abdominal surgery: (I) bundle care: multicomponent, coordinated preventive and nursing programs; (II) anaesthesia interventions: variations in anaesthesia techniques and intraoperative oxygen management; and (III) pharmacological interventions: medication administration aimed at delirium prevention.	Multimodal interventions can reduce postoperative delirium by 49%, with risk influenced by surgical technique and anaesthesia type. Clear communication, family involvement, standardised protocols, and a multidisciplinary approach-including early risk identification and combined nonpharmacological and pharmacological strategies-are key to effective prevention and treatment.	Nurses are part of a multidisciplinary team that works together to care for patients and can encourage families to cooperate.	The authors conducted an analysis of the heterogeneity of results, pointing to significant differences between studies. A subgroup analysis indicated a greater effect of the intervention in smaller studies, which were characterised by a higher risk of research errors. It should be noted that the authors did not perform a formal assessment of the risk of research errors in individual studies, which may limit the interpretation of the results.
32.	[60]	Patients from different wards	Meta-analysis (32 included studies)	Intervention carried out by nurses	Multicomponent nonpharmacological interventions effectively reduce the incidence of delirium in hospitalised patients and mortality, but do not significantly affect its duration, severity, or length of hospitalisation.	Nurses effectively improve treatment outcomes by using nonpharmacological multicomponent interventions, but this can increase the workload of staff.	The included studies were heterogeneous in interventions, patient populations, and outcomes, limiting data synthesis. Risk of bias was assessed using the Cochrane Risk of Bias Tool 2.0, but specific biases were not detailed. Publication bias was not explicitly addressed, potentially overestimating intervention effectiveness.
33.	[10]	Elderly patients in the ICU	Systematic review (14 included studies)	Delirium management and prevention	Pharmacological interventions have been identified as viable pharmacological options for reducing the incidence of delirium. Bright light therapy to improve circadian rhythm and sleep/wake cycles, and the combination of epidural and general anaesthesia was effective, as were nonpharmacological and mixed interventions.	As part of nonpharmacological interventions, nurses can use nursing programs that focus on optimising modifiable risk factors and using therapies such as bright light therapy.	The authors point to significant heterogeneity in the included studies, including differences in diagnostic tools, populations, and age criteria, which makes it difficult to compare results. In addition, the number of studies on nonpharmacological interventions is limited, and their samples are small, which reduces the strength of the evidence. Another significant limitation is the lack of analysis of the effectiveness of interventions in relation to different types of delirium.
34.	[61]	Patients in critical condition	Systematic review and meta-analysis (14 included studies)	Sensory interventions	Sensory interventions, particularly auditory stimulation, can help reduce the risk of delirium in critically ill ICU patients, although overall sensory-based strategies show limited effectiveness in prevention.	When choosing sensory interventions, nurses should prefer auditory stimulation.	The study did not provide a detailed assessment of methodological limitations or risk of bias. Potential limitations include the lack of randomisation and a control group, absence of blinding, single-center design limiting generalisability, no evaluation of long-term outcomes (e.g., cognitive function or quality of life), and unaddressed potential for publication bias.
35.	[62]	Coronary ICU patients (N = 44)/Turkey	Original paper	Nurse-led circadian rhythm–based care: scheduling care to minimise nighttime disturbances, noise control, and use of earplugs to improve sleep, reduce pain and anxiety, and prevent delirium.	Nursing care should be adapted to the patient’s natural circadian rhythm, provided at the right time, and focused on improving sleep quality, as sleep problems can cause pain, anxiety, and delirium.	Nurses caring for patients in accordance with the sleep–wake cycle and assessing the need for intervention effectively improve sleep quality, reducing pain, anxiety, and delirium in patients receiving intensive cardiac care.	Single-center study with a small sample size. Potential confounding factors, such as medications and comorbidities, were not controlled. Reliance on self-reported measures of sleep quality and anxiety may introduce measurement bias.
36.	[63]	Patients in critical condition	Meta-analysis (11 included studies)	Nonpharmacological interventions	Multicomponent interventions significantly reduced the incidence of delirium in critically ill patients. These include: sleep promotion (SP): noise and light control, minimisation of night care; cognitive stimulation (CS): orientation of patients to date, time, and place; hearing and visual acuity support; music, word games, crossword puzzles, and board games; early mobilisation (EM); pain control (PC); and assessment.	Nurses are an integral part of the intensive care team and play a key role in the multidisciplinary team implementing multicomponent interventions to prevent delirium, particularly in interventions requiring specialist knowledge and skills.	Studies included small sample sizes, showed variable quality in reviews and meta-analyses, lacked blinding and randomisation in some cases, raised risk of bias concerns, and did not provide direct head-to-head comparisons.
37.	[64]	Elderly patients with femoral neck fractures (N = 174)/China	Original paper	A multi-component interdisciplinary program	A nurse-led, multicomponent interdisciplinary program can effectively reduce the incidence of postoperative delirium and postoperative hypoxia in elderly patients with femoral neck fractures. However, it does not appear to significantly affect the severity or duration of delirium, nor the overall length of hospital stay.	An interdisciplinary, multi-component perioperative program led by nurses is feasible and effective in reducing the incidence of postoperative delirium in elderly patients with hip fractures, indicating the important role of nurses in preventing delirium.	The study did not provide a detailed assessment of methodological limitations or risk of bias. Potential limitations include the lack of randomisation and a control group, absence of blinding, single-center design limiting generalisability, no evaluation of long-term outcomes (e.g., cognitive function or quality of life), and unaddressed potential for publication bias.
38.	[65]	Patients with femoral neck fractures (N = 41)/Turkey	Original paper	Delirium prevention protocol	Implementing a multicomponent care protocol- including vital signs monitoring, oxygen support for saturation below 90%, nutrition and hydration assessment, and patient orientation-can improve patient outcomes and support delirium prevention.	Nurses play a central role in preventing delirium by identifying risk factors and managing at-risk patients according to established protocols. Their early recognition and holistic care are essential for timely intervention. Training in nonpharmacological strategies to improve sleep and manage delirium risk factors, combined with collaboration with geriatric units, supports a comprehensive approach to patient care.	Single-researcher study with patient assessments limited to twice daily. Although the geriatrician was blinded to group assignments, the evaluation process may still have caused bias. The protocol is specific to older patients with hip fractures, limiting generalisability to other age groups or conditions.
39.	[66]	Emergency department patients (N = 3383)/United States	Original paper	Practical interventions. Evaluation of factors affecting delirium duration.	Bladder catheterisation in elderly patients in the emergency department is associated with a longer duration of delirium.	Nurses should minimise the duration of urinary catheter use in elderly patients in the emergency department, as prolonged catheter use is associated with prolonged delirium.	The study was conducted at a single academic center. It did not assess long-term outcomes, such as cognitive function or mortality, due to the limited sample size.

Abbreviations: 4-AT-Alertness, Abbreviated Mental Test-4, Attention, Acute change or fluctuating course test; ABCDEF bundle-Assess, prevent, and manage pain, Both spontaneous awakening and breathing trials, Choice of analgesia and sedation, Delirium: assess, prevent, and manage, Early mobility and exercise, Family engagement and empowerment; CAM-ICU—Confusion Assessment Method for the Intensive Care Unit; CHI—cerebral hemodynamics improvement; CS—cognitive stimulation; DSM-5—Diagnostic and Statistical Manual of Mental Disorders, 5th Edition; Dy-Del—Dynamic Delirium intervention; EM—early mobilisation; EP—exercise program; FP—family participation; ICU—intensive care unit; KAP—Knowledge, Attitudes, Practices; MLT—multicomponent training; M.O.R.E.—Music, Opening of blinds, reorientation, eye/ear protocols; PC—pain control; PEI—physical environment intervention; RCT—randomised controlled trial; SP—sleep promotion; SR—sedation reduction; UC—usual care.

**Table 3:** Assigning publications to specific subject areas.

Reference	Nursing Role	Intervention Type	Clinical Setting	Effect Direction
[10]	Delirium management	Nonpharmacological and pharmacological	ICU	positive
[11]	Delirium diagnosis	Delirium diagnosis	non-ICU	positive
[12]	Delirium diagnosis	Delirium diagnosis	ICU	positive
[13]	Delirium management and preventive interventions	Nonpharmacological, pharmacological and multicomponent	non-ICU	positive
[14]	Preventive interventions	Nonpharmacological	non-ICU	positive
[18]	Delirium diagnosis	Delirium diagnosis	non-ICU	positive
[21]	Delirium diagnosis	Delirium diagnosis	ICU	positive
[23]	Delirium management	Nonpharmacological	ICU	positive
[36]	Preventive interventions	Nonpharmacological	non-ICU	null
[37]	Delirium management and preventive interventions	Multicomponent	ICU	positive
[38]	Preventive interventions	Multicomponent, nonpharmacological and pharmacological	non-ICU	positive
[39]	Delirium management and diagnosis	Nonpharmacological	non-ICU	null
[40]	Preventive interventions	Nonpharmacological and pharmacological	ICU	null
[41]	Delirium diagnosis	Nonpharmacological and pharmacological	ICU	positive
[42]	Preventive interventions	Nonpharmacological	non-ICU	positive
[43]	Delirium management and preventive interventions	Nonpharmacological and multicomponent	non-ICU	positive
[44]	Delirium management and preventive interventions	Nonpharmacological and multicomponent	ICU	positive
[45]	Preventive interventions	Nonpharmacological	ICU	null
[46]	Delirium diagnosis	Nonpharmacological and multicomponent	ICU	negative
[47]	Preventive interventions	Nonpharmacological	ICU	null
[48]	Preventive interventions	Multicomponent	non-ICU	negative
[49]	Preventive interventions	Nonpharmacological	ICU	positive
[50]	Preventive interventions	Nonpharmacological, multicomponent	ICU	positive
[51]	Delirium management	Nonpharmacological	ICU	positive
[52]	Delirium management	Nonpharmacological	ICU	positive
[53]	Preventive interventions	Nonpharmacological, multicomponent	ICU	positive
[54]	Preventive interventions	Nonpharmacological	ICU	positive
[55]	Preventive interventions	Nonpharmacological	ICU	null
[56]	Preventive interventions	Nonpharmacological	ICU	positive
[57]	Delirium diagnosis	Delirium diagnosis and care for delirium patients	ICU	positive
[58]	Preventive interventions	Nonpharmacological	ICU	positive
[59]	Preventive interventions	Multicomponent	non-ICU	positive
[60]	Delirium management and diagnosis	Nonpharmacological, multicomponent	non-ICU	positive
[61]	Preventive interventions	Nonpharmacological	non-ICU	positive
[62]	Delirium management and preventive interventions	Nonpharmacological	ICU	positive
[63]	Delirium management and preventive interventions	Nonpharmacological, multicomponent	ICU	positive
[64]	Preventive interventions	Multicomponent	non-ICU	positive
[65]	Preventive interventions	Multicomponent	non-ICU	positive
[66]	Delirium diagnosis	Nonpharmacological	non-ICU	null

Abbreviation: ICU—intensive care unit.

## Data Availability

Data are contained within the article.

## Supplementary Materials

The following supporting information can be downloaded at: https://www.mdpi.com/article/10.3390/jcm14228113/s1, Table S1: The PRISMA checklist [95].

## Abbreviations

The following abbreviations are used in this manuscript:

ABCDEF bundle	Assess, prevent, and manage pain, Both spontaneous awakening and breathing trials, Choice of analgesia and sedation, Delirium: assess, prevent, and manage, Early mobility and exercise, Family engagement and empowerment
AS	Assessment strategy
4AT	Alertness, Abbreviated Mental Test-4, Attention, Acute change or fluctuating course Test
CA	Cardiac arrest
CAM	Confusion Assessment Method
CAM-ICU	Confusion Assessment Method for the Intensive Care Unit
CHI	Cerebral hemodynamics improvement
CS	Cognitive stimulation
DSM-5	Diagnostic and Statistical Manual of Mental Disorders, 5th Edition
Dy-Del intervention	Dynamic Delirium intervention
EM	Early mobilization
EP	Exercise program
FP	Family participation
HELP	Hospital Elder Life Program
ICD-10/ICD-11	International Classification of Diseases, 10th/11th revision
ICDSC	Intensive Care Delirium Screening Checklist
ICUs	Intensive care units
KAP	Knowledge, Attitudes, Practices
M.O.R.E.	Music, Opening of blinds, Reorientation, Eye/Ear protocols
MLT	Multicomponent training
NICE	National Institute for Health and Care Excellence
Nu-DESC	Nursing Delirium Screening Scale
PADIS guidelines	Pain, Agitation/Sedation, Delirium, Immobility, and Sleep Guidelines
PC	Pain control
PEI	Physical environment intervention
PRISMA	Preferred Reporting Items for Systematic Reviews and Meta-Analyses
RCTs	Randomized controlled trials
SP	Sleep promotion
SR	Sedation reduction
UC	Usual care
